# Growth differentiation factor 11 (GDF11) has pronounced effects on skin biology

**DOI:** 10.1371/journal.pone.0218035

**Published:** 2019-06-10

**Authors:** Jolanta Idkowiak-Baldys, Uma Santhanam, Sean M. Buchanan, Kathleen Lindahl Pfaff, Lee L. Rubin, John Lyga

**Affiliations:** 1 Global Innovation Center, Avon Products Inc., Suffern, NY, United States of America; 2 Department of Stem Cell and Regenerative Biology, Harvard University, Cambridge, MA, United States of America; Medical University of South Carolina, UNITED STATES

## Abstract

Growth differentiation factor 11 (GDF11) belongs to the TGF-β superfamily of proteins and is closely related to myostatin. Recent findings show that GDF11 has rejuvenating properties with pronounced effects on the cardiovascular system, brain, skeletal muscle, and skeleton in mice. Several human studies were also conducted, some implicating decreasing levels of circulating GDF11 with age. To date, however, there have not been any reports on its role in human skin. This study examined the impact of GDF11 on human skin, specifically related to skin aging. The effect of recombinant GDF11 on the function of various skin cells was examined in human epidermal keratinocytes, dermal fibroblasts, melanocytes, dermal microvascular endothelial cells and 3D skin equivalents, as well as in *ex vivo* human skin explants. GDF11 had significant effects on the production of dermal matrix components in multiple skin models *in vitro* and *ex vivo*. In addition, it had a pronounced effect on expression of multiple skin related genes in full thickness 3D skin equivalents. This work, for the first time, demonstrates an important role for GDF11 in skin biology and a potential impact on skin health and aging.

## Introduction

Aging is defined as the progressive accumulation of diverse deleterious changes in cells and tissues with advancing age that increase the risk of disease and death. Not surprisingly, there is considerable interest in delaying this progression, and research aimed at understanding the aging process and identifying treatments that might slow or even reverse age-related changes is a burgeoning field.

Growth differentiation factor 11 (GDF11), a member of the activin/transforming growth factor-β (TGF-β) superfamily of growth and differentiation factors, came to prominence in aging research as the result of a study that showed levels of this circulating protein were decreased in old compared to young mice [1]. Importantly, the authors found that intraperitoneal injection of GDF11 reversed age-related cardiac hypertrophy in old mice [1, 2]. Restorative effects for GDF11 were also reported in mouse skeletal muscle [3], bone [4], brain [5, 6] and kidney [7]. A study conducted in humans found that levels of GDF11 decreased with age, and reported an association between higher plasma levels of GDF11, and its homolog GDF8, with a reduced risk of cardiac events and death [8]. However, studies that ascribe a positive effect on longevity to GDF11 are not without criticism as some have even proposed GDF11 as a risk factor for certain age-related conditions [9–12]. There remains considerable evidence that suggests a potential rejuvenating role for GDF11 in some organs and tissues.

Skin is the body’s interface with the external environment, and in addition to performing numerous functions that are essential for maintaining life, it also can provide visual information about an individual’s health and age. To the latter point, changes in skin texture, pigmentation, and the presence of lines and wrinkles are commonly observed signs of visual skin aging. A slowdown of epidermal turnover, decrease in production and increased degradation of dermal matrix components (such as hyaluronic acid, collagen) as well as dysregulation of melanin production resulting in uneven pigmentation are among major reasons of the skin changes observed during aging [13]. These signs begin to appear as early as the thirties, but their onset and progression are accelerated by extrinsic factors such as UV exposure. Topical anti-aging products are often a first-line treatment choice to help counter visible signs of aging and maintain a youthful appearance.

A number of recent studies report that autologous platelet rich plasma (PRP) has the potential to improve skin structure and appearance [14–16]. Some of the beneficial effects of this treatment are attributed to various growth factors through their abilities to boost cellular proliferation and stimulate fibroblasts to produce collagen, hyaluronic acid, and other proteins that compose the extracellular matrix [15]. There are also reports indicating that topical application of TGF improves facial wrinkles and other signs of photodamage [14, 17]. As noted, GDF11 belongs to the TGF-β superfamily of growth and differentiation factors. Given the positive anti-aging effects reported for TGF on the skin, and the age-reversing effect reported for circulating GDF11 on certain tissues and organs, we asked whether GDF11 might also provide an anti-aging benefit to skin. To our knowledge, this study is the first to postulate a potential role for GDF11 in skin biology and aging.

## Materials and methods

### *In vitro* effects of GDF11

The *in vitro* assays and analyses for hyaluronic acid, collagen, and melanin were each performed three or more times.

#### Hyaluronic acid and procollagen production

The effects of GDF11 on procollagen I and hyaluronic acid production were assessed in primary human dermal fibroblasts obtained from a 51-year-old female donor (Cascade Biologics, Portland, OR) and full-thickness 3D tissue skin equivalents (EpiDermFT EFT-400, MatTek, Ashland, MA) (Tables 1 and 2). The latter system was used to mimic GDF11’s systemic effect on skin. Human dermal fibroblasts cell cultures were grown under standard cell culture conditions in DMEM medium supplemented with 10% fetal bovine serum and 1% L-glutamine. For experiment cells were seeded at density of 10,000 cells/0.2ml into each well of a 96-well plate (triplicate for each treatment). After overnight incubation, media was changed into serum and growth factor-free DMEM medium supplemented with 1% L-glutamine and 0.0005% ascorbic acid. Cells were treated for 48h with 10 ng/ml or 100 ng/ml recombinant GDF11 (rGDF11, R&D Systems, Minneapolis, MN). The rGDF11 concentrations were chosen to approximate GDF11 levels reported for human sera [9]. Conditioned medium from the fibroblast cultures was collected after 48 hours and stored at -80°C.

**Table 1:** Effect of rGDF11 on procollagen I production in primary human dermal fibroblast culture and full-thickness skin equivalents.

System/rGDF11 Concentration	Mean Percentage Change (p-value)^1^	Concentration ng/mL (SD)
**Primary human dermal fibroblasts**		
Vehicle		476.03 (±62.81)
10 ng/ml	24.0 (0.002)	579.26 (±49.54)
100 ng/ml	49.9 (0.004)	700.25 (±82.29)
**EpiDermFT skin equivalents**		
Vehicle		294.76 (±39.01)
10 ng/ml	8.2 (0.080)	318.8 (±36.96)
100 ng/ml	26.9 (<0.001)	374.2 (±39.01)

^1^Compared to vehicle control. Comparative statistics were performed as two-tailed t-test.

**Table 2:** Effect of rGDF11 on hyaluronic acid production in primary human dermal fibroblast culture and full-thickness skin equivalents.

System/rGDF11 Concentration	Mean Percentage Change (p-value)^1^	Concentration ng/mL (SD)
**Primary human dermal fibroblasts**		
Vehicle		559.66 (±74.51)
10 ng/ml	35.5 (0.002)	798.44 (±59.69)
100 ng/ml	33.2 (0.011)	731.96 (±13.6)
**EpiDermFT skin equivalents**		
Vehicle		560.13 (±120.63)
10 ng/ml	37.9 (0.002)	722.56 (±52.69)
100 ng/ml	6.7 (0.660)	597.75 (±164.11)

^1^Compared to vehicle control. Comparative statistics were performed as two-tailed t-test.

Skin equivalents were grown for a total of 48 hours in proprietary growth factor-free media provided by the manufacturer. rGDF11 was applied into the culture media for 24 h, followed by collection of media and reapplication of fresh media containing rGDF11 for another 24h. Two 24-hour conditioned medium fractions from each culture were pooled and stored at -80°C. Frozen samples were thawed and analyzed by homogeneous time resolved fluorescence (HTRF, Cisbio, Bedford, MA) using anti-human procollagen I antibodies or hyaluronic acid binding protein provided by the kit. 20ng/mL of TGFβII was used as assay positive control for pro-collagen I production and 1ng/mL of IL-1β as positive control for HA production in both models.

#### Melanin production

B16 mouse melanoma cells (ATTC, Manassas, VA) were grown under standard cell culture conditions in DMEM medium supplemented with 10% fetal bovine serum. For experiments, cells were seeded at density of 20,000 cells/0.2ml into each well of a 96-well plate (six replicates for each treatment). Before treatment, cells were transferred to serum and growth factor-free medium supplemented with 2nM of α-MSH and treated with 10 ng/ml or 100 ng/ml rGDF11. After the 3 days of incubation, when a visible change in the color of the medium should be apparent, the medium was collected, and its absorbance measured at 540 nm to assess melanin production (Table 3).

**Table 3:** Effect of rGDF11 on melanin production in mouse melanocyte culture and pigmented human skin equivalents.

System/rGDF11 Concentration	Mean Percentage Change (p-value)^1^
**Mouse B16 melanoma cells**	
10 ng/ml	-24.0 (<0.001)
100 ng/ml	-30.0 (<0.001)
**MelanoDerm skin equivalents**	
20 ng/ml	-46.2 (<0.001)
200 ng/ml	-31.7 (0.001)

^1^Compared to vehicle control. Comparative statistics were performed as two-tailed t-test.

Pigmented 3D tissue skin equivalents (MelanoDerm MEL300-B, MatTek) were grown in growth factor-free medium (provided by manufacturer) supplemented with 20 ng/ml or 200 ng/ml rGDF11. Tissues were treated for total of 14 days with fresh media and treatment added every 2 days. After 14 days, melanin was extracted from the tissue with Solvable (PerkinElmer, Waltham, MA) and analyzed by a standard MatTek protocol (Table 3). Briefly, each tissue is removed from the insert and transferred into a tube containing 500μM Solvable and incubated at 95°C overnight along with melanin standards. After incubation, samples were spun at 13000rpm and 300μL of supernatant containing dissolved melanin was transferred to microwell plate. Plate reading was done at 490nM.Kojic acid (0.1%) was used as a positive control in both systems.

#### Gene expression analysis

EpiDermFT EFT-400 skin equivalents were treated for 24 hours with 100 ng/ml rGDF11. The tissues were then rinsed with PBS, flash-frozen in liquid nitrogen, and stored at -80°C. RNA was extracted using TRIzol (ThermoFisher Scientific, Waltham, Massachusetts), and 400 ng of total RNA was used for cDNA synthesis using an RT^2^ First Strand Kit (Qiagen, Valencia, CA). The expression of 80 genes relevant to skin biology was analyzed using a custom-designed RT^2^ Profiler PCR Array (Qiagen) (Table 4 and S1 File). Statistical analysis was performed using web-based data analysis provided by Qiagen (https://www.qiagen.com/us/shop/genes-and-pathways/data-analysis-center-overview-page/?akamai-feo=off).

**Table 4:** Effect of rGDF11 on gene expression in full-thickness skin equivalents.

Gene Symbol	Fold Regulation	Skin-Related Gene Function
COL1A1	2.4	Dermal matrix
COL6A6	4.5	Dermal matrix
COL14A1	2.8	Dermal matrix
MMP9	3.5	Dermal matrix
TIMP2	1.5	Dermal matrix
ELN	3.6	Dermal matrix
TGFBR3	3.6	Dermal matrix
HAS1	3.4	Dermal matrix, hydration
ALOX12	3.6	Barrier
ALOX12B	2.6	Barrier
ALOXE3	2.0	Barrier
DSG1	2.2	Barrier
DSP	2.2	Barrier
EZH2	2.4	Proliferation
EZH1	2.8	Proliferation
HBEGF	2.1	Proliferation
KRT6B	2.1	Epidermal health/differentiation
IL1B	-2.2	Inflammation
KLK7	2.0	Epidermal health/turnover

### Human skin explant study

The effect of GDF11 on procollagen I, hyaluronic acid, and melanin in human skin explants was examined in a study conducted at an independent testing facility (Cutech Srl, Padova, Italy).

#### Tissue preparation and treatment

Skin was isolated from surgical leftover residues (waste material destined for destruction) following abdominal plastic surgery performed on a healthy 54-year-old female.

Written informed consent for use of the waste material was obtained in accordance with the Helsinki Declaration. Colorimetric analysis classified the skin as ‘tanned’ (ITA° = 13°, Del Bino S. et al. 2006). Biopsies of approximately 8 x 3 mm (Ø x thickness) were cut from the skin and cultured in an air-liquid interface in modified Williams’ E medium (Sigma-Aldrich) for 6 days for collagen and pigmentation analysis and 3 days for hyaluronic acid analysis. Four microliters of the positive control treatments were applied daily to the surfaces of replicate (n = 8 for each treatment) cultured mounts: 10 μM retinol (positive control for pro-collagen and hyaluronic acid); and 0.1% kojic acid (Sigma-Aldrich) (positive control for pigmentation), while 10 ng/ml or 100 ng/ml GDF11 were added to the culture media to mimic systemic treatment. Replicates were also treated with the corresponding vehicles.

#### Explant viability

After 6 days of incubation, two cultured replicates for each treatment were assessed by viability assay. Briefly, this assay provides an indication of cell viability based on the conversion of 3-(4,5-dimethylthiazol-2-yl)-2,5-diphenyltetrazolium bromide (MTT) to water-insoluble, dark blue-colored MTT-formazan by mitochondrial dehydrogenases that are present in viable cells. The blue crystals formed were solubilized and the color intensity at 570 nm was measured.

#### Skin morphology and potential toxicity evaluation

Twelve skin sections for each treatment from the remaining cultured replicates were evaluated after hematoxylin eosin staining. The stained sections were evaluated and scored for potential toxicity using a 6-point scale: 0 = physiological morphology; 1 = localized dermo/epidermal vacuolization; 2 = epidermal vacuolization and/or mild dermo/epidermal junction loosening; 3 = epidermal vacuolization and/or localized dermo/epidermal detachment; 4 = diffuse dermo/epidermal detachment; 5 = complete compromise of the skin morphology.

#### Procollagen I detection and semi-quantitative analysis

**Fig 1:**
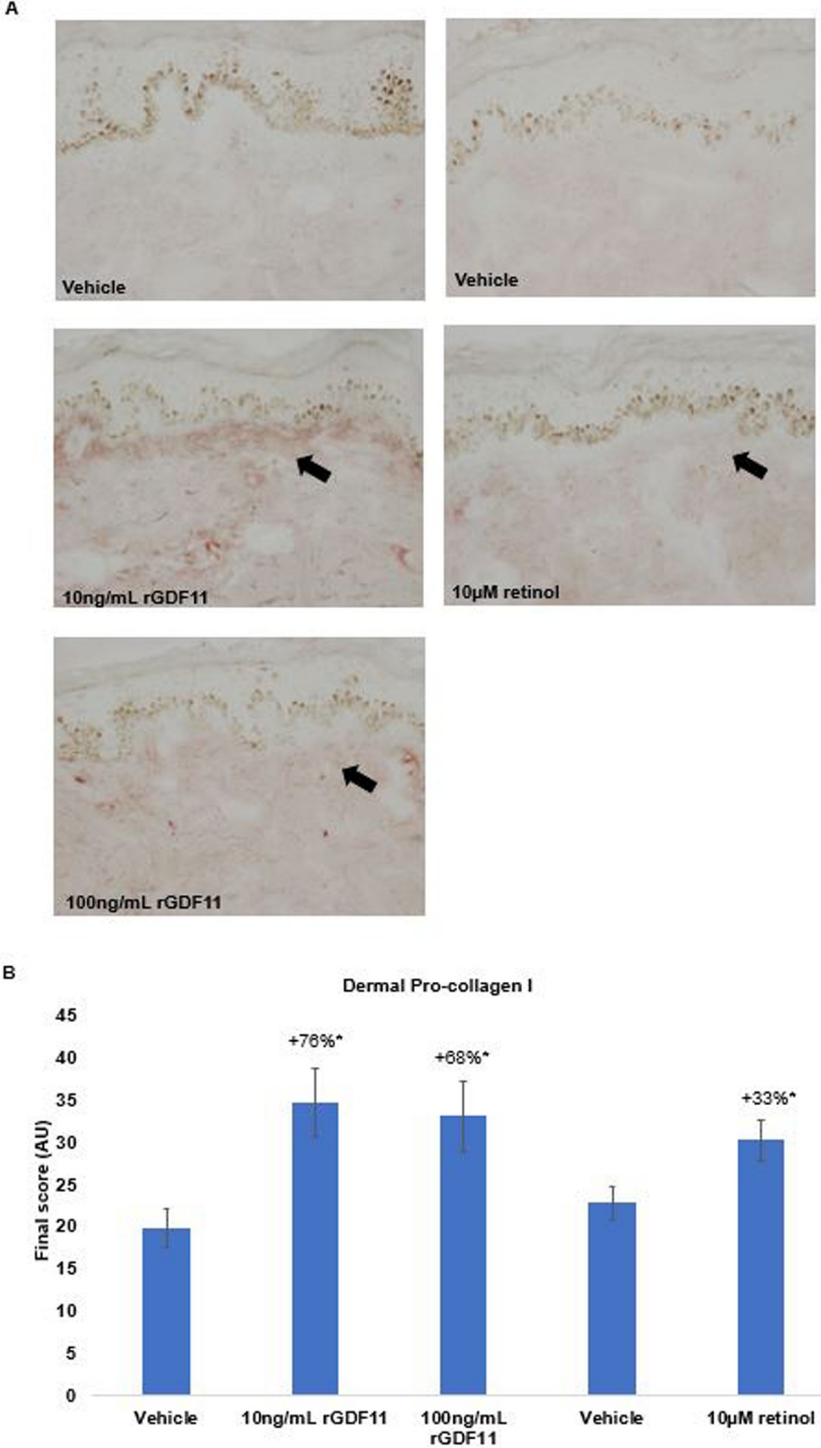
Effect of rGDF11 on procollagen I production in human skin explants. (a) Representative images of dermal procollagen I immunostaining. Replicate (n = 8) skin explants were cultured for 6 days in modified Williams’ E medium and treated daily with rGDF11 (added to the media at concentration of 10 ng/ml or 100 ng/ml), retinol (10 μM, topical 4μL), or corresponding vehicle. Twelve sections were immunostained and the amount of procollagen I was determined by image analysis. (b) Comparison of procollagen I scores assigned to sections (n = 12) treated with rGDF11, retinol, or vehicle. Results are shown as the mean +/- SEM; * indicates the change versus vehicle is statistically significant at p ≤0.05. Statistical analysis used: One-way ANOVA with permutation test: F (4,55) 4.35; p-value <0.01, followed by Tukey’s permutation test.

Twelve skin sections for each treatment were immunostained using procollagen I antibody (ab64409, Abcam, Cambridge, MA). The amount of procollagen I was evaluated based on the intensity and distribution of immunostained procollagen I in the area corresponding to the papillary dermis, just below the epidermis. As above, 8-bit grey-scale photomicrographs were captured and transformed from RGB to the standard CIE L*a*b* color space. The L* value for each pixel in the ROI was evaluated using ImageJ software (National Institutes of Health, Bethesda, MD) with the Color Inspector 3D plug-in. Scores reflecting the amount of procollagen I detected were assigned using an image analysis algorithm proprietary to Cutech Srl (Fig 1).

#### Dermal hyaluronic acid detection and semi-quantitative analysis

**Fig 2:**
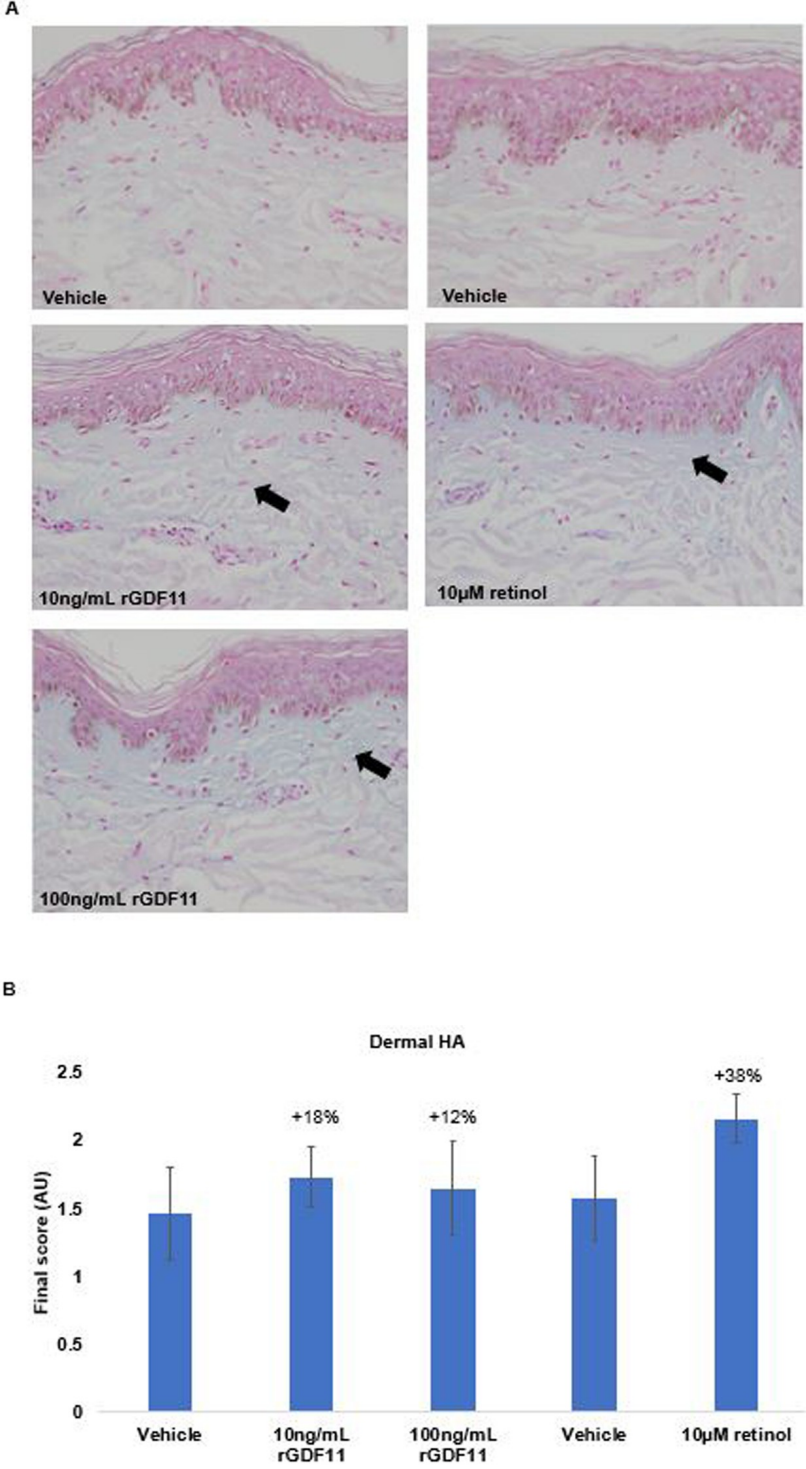
Effect of rGDF11 on hyaluronic acid production in human skin explants. (a) Representative images of hyaluronic acid staining. Replicate (n = 8) skin explants were cultured for 3 days in modified Williams’ E medium and were treated daily with rGDF (added to the media at concentration of 10 ng/ml or 100 ng/ml), retinol (10 μM topical 4μL), or corresponding vehicle. Twelve sections were stained with alcian blue stain and the amount of hyaluronic acid was determined by image analysis. (b) Comparison of hyaluronic acid scores assigned to sections (n = 12) treated with rGDF11, retinol, or vehicle. Results are shown as the mean +/- SEM. Statistical analysis used: One-way ANOVA with permutation test: F (4,55) 0.85; p-value >0.05, followed by Tukey’s permutation test.

Twelve skin sections for each treatment were stained with alcian blue stain. The amount of hyaluronic acid present was evaluated based on the intensity and distribution of blue color in the dermis using an imaging method analogous to that used for procollagen I. Scores reflecting the amount of hyaluronan detected were assigned using an image analysis algorithm proprietary to Cutech Srl (Fig 2).

#### Melanin detection and semi-quantitative analysis

Twelve skin sections for each treatment were stained with Fontana-Masson stain. The amount of melanin present was determined by estimating the grey level intensity and distribution in the stained sections. Briefly, 8-bit, grey-scale photomicrographs were captured and transformed from RGB to the standard CIE L*a*b* color space. L* values were measured at each pixel to express the luminance or level of grey from black (value = 0) to white (value = 100). The results were rank-transformed then normalized based on the ratio of values for a selected region of interest (ROI) to values for the total area of the slide. Scores reflecting the amount of melanin detected were assigned using an image analysis algorithm proprietary to Cutech Srl (S1 Fig).

Overview of all experimental procedures is summarized in S1 Table.

### Smad2/3 pathway activation

**Fig 3:**
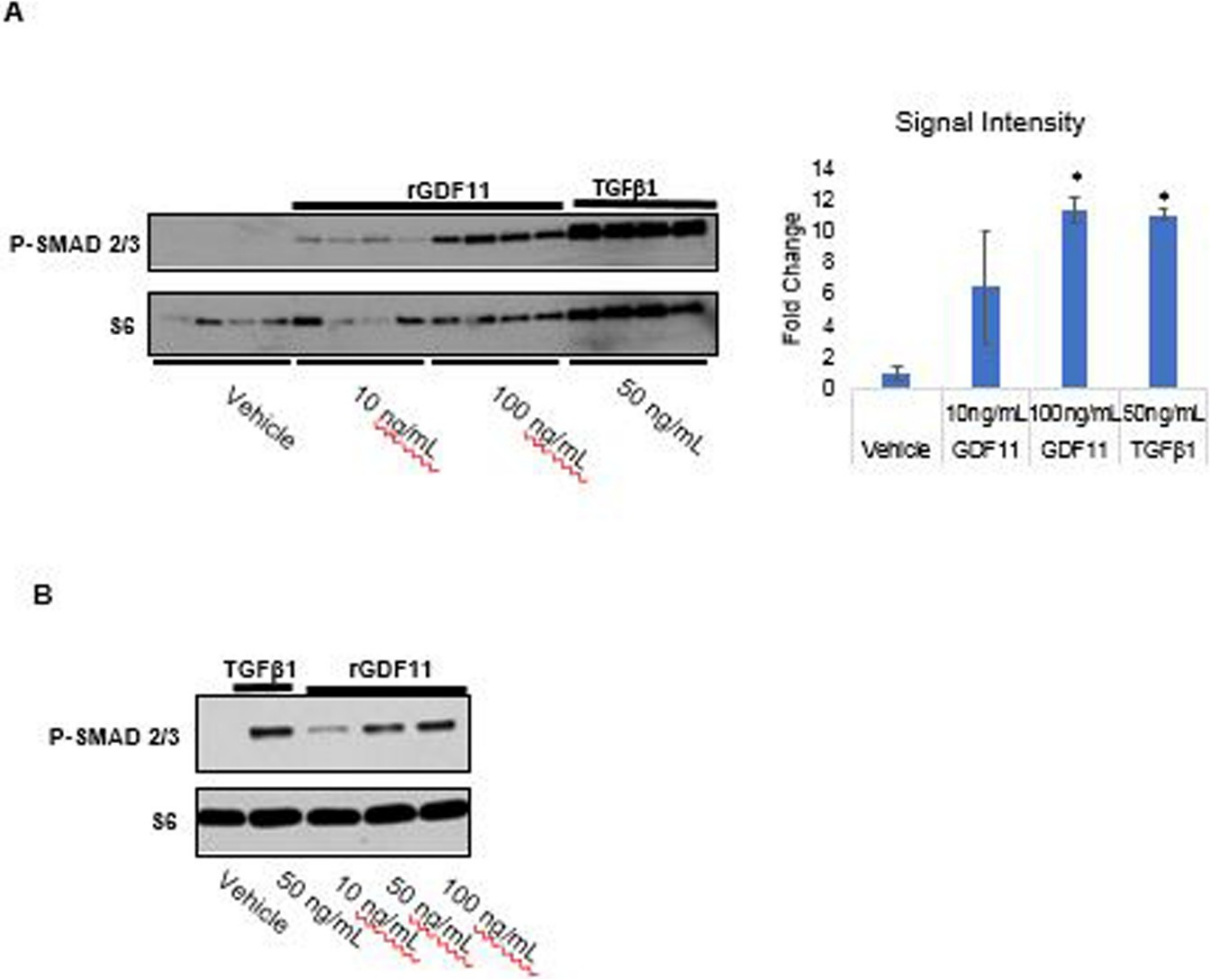
rGDF11 activates Smad2/3 signaling in human skin models. (a) Human full thickness skin equivalents were treated with vehicle, rGDF11 (10ng/ml or 100ng/ml), or TGF-β1 (50ng/ml) for 2 hours, lysed, and Smad2/3 phosphorylation was analyzed by immunoblotting. Ribosomal protein S6 was used as a loading control. Semiquantitative analysis of signal intensity was performed on the original TIFFs files in Photoshop. Background-subtracted signal was normalized to vehicle treatment and converted to a fold change. * represents p<0.05 as calculated by T-test. (b) Primary human dermal microvascular endothelial cells were stimulated with rGDF11 (10ng/ml, 50ng/ml, or 100ng/ml) or TGF-β1 (50ng/ml) for 60 minutes, lysed, and P-Smad2/3 and ribosomal S6 levels were analyzed.

Full-thickness 3D tissue skin equivalents (EpiDermFT, MatTek) were treated with 10 ng/ml and 100 ng/ml rGDF11 or 50 ng/ml TGFβ1 (R&D Systems, Minneapolis, MN) added into proprietary growth factor-free media provided by the manufacturer for 2 hours. The treated tissues were placed on ice, washed with ice-cold PBS, lysed in 600μl T-PER Tissue Protein Extraction Reagent (ThermoFischer Scientific), and reacted with anti-P-Smad3 clone (EP823Y, Abcam, Cambridge, MA) and anti-S6 clone (5G10, Cell Signaling, Danvers, MA). The P-Smad2/3 and ribosomal S6 levels were assessed by western blot analysis. Ribosomal S6 was used as a loading control (Fig 3A). Primary human dermal microvascular endothelial cells (HDMEC, PromoCell, Heidelberg, Germany) were grown in Endothelial Growth Cell Medium MV with supplements (PromoCell, Heidelberg, Germany) and seeded for experiment at density of 100,000 cells/5ml into each well of a 6-well plate (triplicate for each treatment). After overnight incubation, cells were transferred into media without supplement and treated with 10 ng/ml, 50 ng/ml, and 100 ng/ml rGDF11 or 50 ng/ml TGFβ1 for 60 minutes. The treated cells were placed on ice, washed with ice-cold PBS, and lysed in 200 μl M-PER Mammalian Protein Extraction Reagent (ThermoFischer Scientific). The P-Smad2/3 and ribosomal S6 levels were analyzed as above (Fig 3B).

## Results

### Recombinant GDF11 leads to induction of extracellular matrix components in skin models

Treating human primary dermal fibroblasts monolayers from an aged donor with 10 ng/ml and 100 ng/ml rGDF11 for 48 hours significantly increased procollagen I production in a dose-dependent manner compared to vehicle (Table 1). Procollagen I production was also increased when full-thickness 3D skin equivalents were treated under the same conditions; however, only the 100 ng/ml level of rGDF11 reached statistical significance (Table 1)

*Ex vivo* results mirrored those generated *in vitro*; immunostained sections from skin explants treated for 6 days with 10 ng/ml and 100 ng/ml rGDF11 showed increased procollagen I production in the upper dermis (Fig 1). Semi-quantitative scoring confirmed that both concentrations of rGDF11 significantly increased dermal procollagen I compared to vehicle (Fig 1). Retinol, which was used as a positive control in the *ex vivo* work, also stimulated dermal procollagen I production.

Analysis of conditioned media collected from fibroblast monolayers treated with 10 ng/ml or 100 ng/ml rGDF11 for 48 hours showed that hyaluronic acid secretion was significantly increased compared to vehicle (Table 2). Only the 10 ng/ml concentration of rGDF11 significantly increased hyaluronic acid production in similarly-treated full-thickness 3D skin equivalents (Table 2).

Semi-quantitative scoring of skin explants treated with rGDF11 or the retinol control for 3 days showed numerically greater hyaluronic acid production compared to the respective vehicles; however, the increases were not statistically significant (Fig 2).

### Recombinant GDF11 can alter melanin production

Melanin production was significantly reduced compared to vehicle in murine B16 melanoma cells grown in the presence of 10 ng/ml and 100 ng/ml of rGDF11 for 3 days (Table 3). A significant reduction in melanin production was also observed when pigmented 3D skin equivalents were grown in the presence of rGDF11 for 14 days. However, daily application of rGDF11 to the media of skin explants for 6 days had no significant effect on melanin production relative to vehicle (S1 Fig).

### Recombinant GDF11 has a beneficial effect on expression of multiple skin-related genes

Treating EpiDermFT equivalents with 100 ng/ml rGDF11 influenced over a dozen genes relevant to skin function in a direction consistent with its positive effect on skin (Table 4). As expected from the above results, there was a pronounced effect (2-fold change or more) on multiple genes related to extracellular matrix production (COL1A1, COL6A6, COL14A1, ELN, TGFBR3, and HAS1). In addition, GDF11 increased, by at least 2-fold, the expression of 5 genes important for maintaining skin barrier function (ALOX12, ALOX12B, ALOXE3, DSG1, and DSP), 3 genes related to cellular proliferation (EZH2, EZH1, HBEGF), and 2 genes related to epidermal turnover and differentiation (KLK7, KRT6B). Interestingly, treatment with rGDF11 yielded over a 2-fold decrease in the gene related to pro-inflammatory cytokine IL1β (IL1B).

### Recombinant GDF11 activates the Smad2/3 pathway in skin

To understand the mechanism of action of rGDF11 on skin, its effect on activation of P-Smad pathway was studied. Treating full thickness skin equivalents with 10 ng/ml and 100 ng/ml rGDF11 increased P-Smad2/3 protein in a dose dependent manner (Fig 3A). In addition to studying its effects on dermal and epidermal components of skin, we wanted to determine if rGDF11 could have potential effects on skin vasculature. For that purpose, primary human dermal microvascular endothelial cells were treated with 10 ng/ml, 50 ng/ml, and 100 ng/ml rGDF11. The results show that rGDF11 activates the Smad2/3 phosphorylation pathway in skin endothelial cells (Fig 3B) with a potential effect on the skin vasculature.

## Discussion

This paper presents data that support a role for circulating GDF11 as a regulator of skin biology. When different skin models including monolayer, 3D tissue skin equivalents, and surgically removed skin explants were treated with physiologically-relevant levels of rGDF11, significant effects on the production of procollagen I and hyaluronic acid were observed. In addition, a decrease in the production of melanin was also observed in melanocytes and 3D skin equivalents. Treatment of full thickness 3D skin equivalents with rGDF11 led to changes in expression of multiple genes that are linked to several important skin functions. Finally, we showed that GDF11 appears to be acting in skin via the Smad2/3 signaling pathway, consistent with it being a member of the TGF-β family.

The original work that identified GDF11 as a potential “youth factor” in the blood [1] sparked considerable excitement in the aging field and led to multiple follow-up studies. Within just a few years, the role of GDF11 in aging was studied in multiple organs and species. Even though skin aging is an important and visible aspect of human aging, there are no reported studies to understand its role in human skin. Using different skin models, we showed clear effects of rGDF11 on extracellular skin matrix production, although dose effect and magnitude varied, which we attributed to different culture conditions. These effects, however, if proven *in vivo*, could be important in targeting age-associated changes in the dermal matrix. A potential effect on decreasing pigmentation was observed in melanocytes and 3D skin equivalents but was not observed in skin explants. The *ex vivo* system might be well-suited to demonstrate the effects of direct tyrosinase inhibitors such as kojic acid. However, it might not be sensitive to effects of growth factors such as GDF11, which could inhibit melanin production via an effect on upstream signaling pathways. It also needs to be noted that results from our *ex vivo* studies are limited to the response from a single donor subjects from which skin samples were obtained.

To better understand the potential effect of GDF11 on skin, the expression of multiple skin-related genes was analyzed in 3D skin equivalents treated with rGDF11. Although we expected some effects, the fact that rGDF11 changed the expression of 19 skin-related genes by at least two-fold was surprising. Positive effects on skin would be predicted for 18 of these genes. Only a change in the expression of collagenase MMP-9, which increased by about 3.5-fold, is potentially detrimental as it could lead to greater matrix degradation. However, since other matrix-related genes were upregulated, we hypothesize that the increase in MMP-9 expression might be more related to matrix remodeling than degradation. It is known that MMP-9 is upregulated during wound healing [18] and interacts with TGF-ß1 to facilitate wound closure [19]. In addition to genes supporting dermal matrix, rGDF11 demonstrated effects on genes related to barrier improvement and proliferation. It also downregulated the pro-inflammatory cytokine IL-1β, which is implicated as one of the factors in skin inflammaging [20, 21].

Since GDF11 belongs to the TGF-β family of proteins it was not surprising that its ability to signal via phosphorylation of Smad2/3 was confirmed in skin models studied. Activation of the Smad2/3 pathway by TGF-β plays an important role in angiogenesis by promoting endothelial cells migration and proliferation [22]. Since this pathway was also shown to be important in GDF11’s effect on brain vasculature [5, 6], we wondered if GDF11 might also have an effect on blood vessels in skin. Since 3D skin equivalents do not have a vascular component, we examined the effect of rGDF11 on dermal microvascular endothelial cells. This study confirmed that rGDF11 induces Smad2/3 phosphorylation in those cells, consistent with possible benefits on skin vasculature, which is known to be impaired by aging [23]. In addition to the effect on endothelial cells it is highly likely that GDF11 may signal via the same pathway in other skin cells such as fibroblasts and keratinocytes to induce the effects we observed on matrix proteins and gene expression.

Aging is a complex process and GDF11 is potentially an important piece of the puzzle. It might have different degrees of importance for different organs and tissues, but our work herein shows its potentially beneficial role in skin biology.

## Supporting information

S1 Fig Effect of rGDF11 on pigmentation in human skin explants. Twelve skin sections for each treatment were stained with Fontana-Masson stain. The amount of melanin present was determined by estimating the grey level intensity and distribution in the stained sections. Scores reflecting the amount of melanin detected were assigned using an image analysis algorithm proprietary to Cutech Srl. *p<0.05. Statistical analysis used: One-way ANOVA with permutation test: F(4.45) 1.87; p-value>0.05, followed by Tukey’s permutation test.
(TIF)

S1 File Gene expression analysis results. (XLS)

S1 Table Summary of skin models and endpoints tested. (DOCX)
