# Supplementary figures and images for: Growth differentiation factor 11 (GDF11) has pronounced effects on skin biology
Source: PLoS One. 2019 Jun 10;14(6):e0218035. doi: 10.1371/journal.pone.0218035 (PMC6557520; doi:10.1371/journal.pone.0218035)

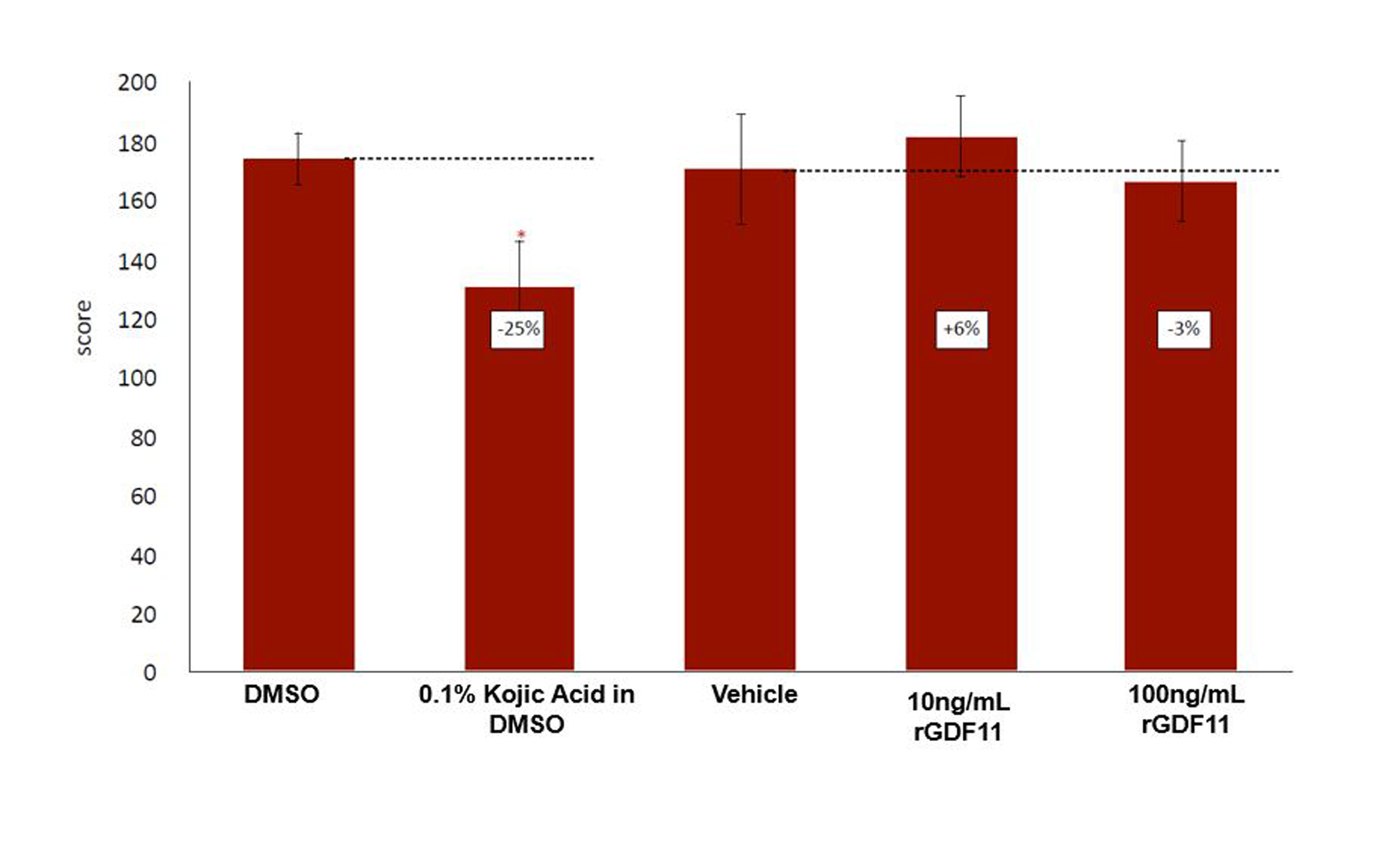

Supplement: S1 Fig — Twelve skin sections for each treatment were stained with Fontana-Masson stain. The amount of melanin present was determined by estimating the grey level intensity and distribution in the stained sections. Scores reflecting the amount of melanin detected were assigned using an image analysis algorithm proprietary to Cutech Srl. *p<0.05. Statistical analysis used: One-way ANOVA with permutation test: F(4.45) 1.87; p-value>0.05, followed by Tukey’s permutation test. (TIF) [file pone.0218035.s001.tif]
