# Supplementary material for: Growth differentiation factor 11 (GDF11) has pronounced effects on skin biology
Source: PLoS One. 2019 Jun 10;14(6):e0218035. doi: 10.1371/journal.pone.0218035 (PMC6557520; doi:10.1371/journal.pone.0218035)
Supplement: S1 Table — (DOCX) [file pone.0218035.s003.docx]

| **Skin Related Endpoint** | **Skin model** | **Assay** | **Effect** |
| --- | --- | --- | --- |
| **Hyaluronic Acid** | Human Dermal Fibroblasts | homogeneous time resolved fluorescence of HA secreted to the media (HABP) | increase |
|  | Full thickness 3D tissues | homogeneous time resolved fluorescence of HA secreted to the media (HABP) | increase |
|  | Skin explants | Histological evaluation of dermal HA using alcian blue stain | increase |
|  |  |  |  |
| **Pro-collagen I** | Human Dermal Fibroblasts | homogeneous time resolved fluorescence of pro-collagen I secreted to the media (anti-pro-collagen I antibody) | Increase |
|  | Full thickness 3D tissues | homogeneous time resolved fluorescence of procollagen I secreted to the media (anti-pro-collagen I antibody) | Increase |
|  | Skin explants | Histological evaluation of dermal procollagen I using anti-procollagen I antibody | increase |
|  |  |  |  |
| **Melanin** | Mouse Melanoma cells | Colorimetric detection of melanin in the media | decrease |
|  | Melanoderm 3D tissues | Colorimetric detection of melanin extracted from tissues | decrease |
|  | Skin explants | Histological evaluation (Fontana Mason staining) | No effect |
|  |  |  |  |
| **Smad2/3 pathway** | Full thickness 3D tissues | Cell Lysate Western blot analysis of Smad2/3 phosphorylation | Increase |
|  | Primary human dermal microvascular endothelial cells | Tissue Lysate Western blot analysis of Smad2/3 phosphorylation | Increase |
|  |  |  |  |
| **Gene Expression** | Full thickness 3D tissues | RT^2^ Profiler PCR Array | Induction of changes in gene expression |
